# Isolation, Characterization, and Preliminary Application of Staphylococcal Bacteriophages in Sichuan *Paocai* Fermentation

**DOI:** 10.3390/microorganisms13061273

**Published:** 2025-05-30

**Authors:** Xia Lin, Chunhui Deng, Luya Wang, Yue Shu, Shengshuai Li, Yunlong Song, Hong Kong, Ziwei Liang, Lei Liu, Yu Rao

**Affiliations:** 1 Food Microbiology Key Laboratory of Sichuan Province, School of Food Science and Bioengineering, Xihua University, Chengdu 610039, China; lxia0924@163.com (X.L.); dengjia991015@163.com (C.D.); yuan8you@163.com (L.W.); shuyuehappy@163.com (Y.S.); leeo0510@outlook.com (S.L.); 19950900744@163.com (Y.S.); kong3053462@163.com (H.K.); ziweiliang1029@outlook.com (Z.L.); 2 State Key Laboratory for Animal Disease Control and Prevention, College of Veterinary Medicine, Lanzhou University, Lanzhou 730000, China

**Keywords:** Sichuan *paocai* fermentation, staphylococcal phages, microbial community structure, lactic acid bacteria

## Abstract

Sichuan *paocai*, a microbial food predominantly fermented by lactic acid bacteria and hosting a complex and diverse microbial ecosystem, serves as an ideal habitat for bacteriophages. However, relatively few studies have been conducted on isolating bacteriophages from fermented vegetables and their application in vegetable fermentation. In this study, three staphylococcal bacteriophages, ΦSx-2, ΦSs-1, and ΦSs-2, were isolated and purified from Sichuan *paocai* using the spot test method. The morphological features of the phages were characterized using transmission electron microscopy (TEM), while key biological properties such as one-step growth kinetics were systematically evaluated, ultimately verifying their taxonomic placement within the Caudoviricetes class. Furthermore, the potential effects of these phages on the microbial community structure and physicochemical properties during *paocai* fermentation were investigated using high-throughput sequencing and standard physicochemical assays. Microbial community analysis demonstrated that introducing the phages significantly increased the relative abundance of lactic acid bacteria while reducing the prevalence of spoilage bacteria such as *Erwinia*, *Pantoea*, and *Enterobacter*. Physicochemical assessments revealed that adding phages accelerated the acidification process of *paocai*, effectively reduced nitrite levels, and increased the concentrations of lactic and acetic acids. Additionally, notable differences in color and flavor were observed between the two groups of *paocai* during the fermentation process. In summary, the inoculation of bacteriophages ΦSx-2, ΦSs-1, and ΦSs-2 optimized the microbial community structure, enhanced the fermentation process, and improved the quality of Sichuan *paocai*.

## 1. Introduction

Bacteriophages (phages) are viruses that specifically infect bacteria. As ubiquitous bacterial predators in nature, they are highly prevalent in fermented foods [1]. Specifically infecting and lysing their bacterial hosts, phages regulate the population structure and quantity of key microorganisms within the fermentation systems [2,3]. These interactions profoundly affect fermented foods’ flavor, texture, and nutritional value [4]. In practical applications, the functional characteristics of bacteriophages have been thoroughly verified during the processing of fermented foods such as Korean kimchi and Chinese vinegar [5,6]. For instance, studies have demonstrated that introducing bacteriophages targeting specific spoilage bacteria during the initial stages of kimchi fermentation can effectively suppress spoilage and significantly reduce nitrate formation [7]. In summary, bacteriophages play a pivotal role in modulating microbial homeostasis during fermentation processes and hold potential as innovative tools for regulating fermentation processes and enhancing product quality.

Sichuan *paocai* is a quintessential example of traditional fermented vegetables, renowned domestically and internationally for its unique flavor, excellent nutritional value, and rich probiotic content. Sichuan *paocai* has become a focus in the field of healthy eating in recent years [8,9]. It holds a prominent position in the Chinese pickled vegetable market, with a market value exceeding RMB 40 billion [10]. The traditional production process of Sichuan *paocai* is relatively straightforward, involving the immersion of fresh vegetables in brine with a salt concentration of 6% to 8%, followed by natural fermentation in a sealed environment [11]. During this relatively short fermentation period, various microbial populations proliferate rapidly, with the maturation process typically lasting from a few days to several weeks or even months [11,12]. The integration of traditional cultivation methods with modern high-throughput sequencing technology has enabled the analysis of dynamic changes in microbial communities during the *paocai* fermentation process. This comprehensive analysis particularly highlights the crucial role of lactic acid bacteria, such as *Lactobacillus* and *Pediococcus,* in driving fermentation [9,13]. While considerable progress has been made in understanding the role of cellular microorganisms in fermentation, research on bacteriophages in traditional Chinese fermented vegetables remains relatively scarce. This paucity of research limits a comprehensive understanding of the macroecology of fermented vegetables. Strengthening research on bacteriophages in traditional Chinese fermented vegetables is, thus, of significant importance, addressing this lack of knowledge in this field while opening new avenues to explore and utilize the potential of phages in fermentation processes.

This study focused on three staphylococcal phages, ΦSx-2, ΦSs-1, and ΦSs-2, isolated from Sichuan *paocai*. Their biological characteristics, including morphology, pH stability, and tolerance to varying salt concentrations, were systematically analyzed. The study also examined their role in shaping the microbial community during *paocai* fermentation and their influence on key physicochemical indicators such as pH, nitrite levels, and organic acid concentrations. Through this study, we aim to deepen our theoretical understanding of the ecological functions of bacteriophages in traditional fermented foods and provide novel application ideas for the development of new technologies related to fermentation process optimization and nitrite control based on bacteriophage regulation.

## 2. Materials and Methods

### 2.1. Preparation of Sichuan Paocai

The specific preparation process of Sichuan *paocai* used for bacteriophage isolation in this study was conducted as follows. Fresh radishes and seasonal vegetables purchased from local markets in Chengdu, Sichuan Province, China, were thoroughly washed with tap water and air-dried until no surface moisture remained before being cut into uniform pieces (approximately 4 cm in length). A 6% (*w*/*v*) NaCl solution was prepared using distilled water, boiled for 15 min, and cooled to room temperature. The prepared vegetables were packed into fermentation jars, and cooled brine was added at a vegetable-to-brine ratio of 1:3 (*w*/*v*). The jars were sealed with water locks to maintain anaerobic conditions and subjected to natural fermentation at room temperature (20 ± 2 °C) for 12 days. Three parallel fermentation jars were prepared to ensure experimental reproducibility.

### 2.2. Amplification and Concentration of Phages in the Sichuan Paocai

The collected 240 mL of *paocai* brine samples from the first three days of fermentation were combined and then centrifuged at 7155× *g*, 4 °C, for 10 min. The supernatant, potentially containing phages, was stored at 4 °C for subsequent use. The bacterial precipitate was resuspended in sterile physiological saline and added to 400 mL of tryptic soy broth (TSB) supplemented with cycloheximide (10 μg/mL). The mixture was then placed in a shaker at 37 °C to culture the early logarithmic growth phase (OD_600nm_ ≈ 0.3). Subsequently, the supernatant was added to the culture, and the mixed culture was incubated under the same conditions at 37 °C for 24 h to produce the phage amplification solution [14].

The phage amplification solution was centrifuged at 7155× *g*, 4 °C, for 10 min. The resulting bacterial precipitate was resuspended in sterile saline and preserved in glycerol for future use. The supernatant was filtered through a 0.22 µm membrane filter and treated with DNase I (1 µg/mL) and RNase A (1 µg/mL) at 37 °C for 30 min. Sodium chloride (final concentration: 1 mol/L) and polyethylene glycol 8000 (10%, *w*/*v*) were added to the treated phage amplification solution. The sample was incubated at 4 °C for 12 h and then centrifuged at 7155× *g* at 4 °C for 20 min. The resulting precipitate was carefully resuspended in SM buffer (5.8 g NaCl, 2 g MgSO_4_·7H_2_O, 50 mL 1 mol/L Tris-Cl, pH 7.5, dissolved in 1 L of water), ultimately yielding a highly concentrated phage preparation [7].

### 2.3. Isolation of Specific Phages and Host Bacteria

The bacterial precipitate preserved in glycerol by freezing was diluted and spread onto tryptone soy agar (TSA) plates, which were incubated at 37 °C for 12 h. Single colonies were subsequently picked from the TSA plates and inoculated into a 96-well plate, with each well containing 200 μL of TSB. After 12 h of incubation at 37 °C, bacterial suspensions from each well were aspirated and spread onto separate TSA plates. A spot test was performed by applying 10 μL of the phage-concentrated solution onto the bacterial lawn formed on the TSA plates. After overnight incubation at 37 °C, the TSA plates showing positive spot tests indicated the presence of isolated phages. These phage isolates were subjected to two rounds of plaque purification to ensure purity. Glycerol stock solutions of the purified phages and their respective host bacteria were prepared and stored at −80 °C for future use [15].

### 2.4. Identification of Specific Host Bacteria

The 16S rRNA gene from the host bacteria was isolated and amplified using polymerase chain reaction (PCR). The PCR mixture consisted of 13 μL 2 × Taq MasterMix, 1 μL each of the forward primer 27F and reverse primer 1492R, 9 μL ddH_2_O, and 1 μL of the bacterial liquid to be analyzed. The PCR process began with an initial denaturation at 94 °C for 5 min, followed by 30 cycles of denaturation at 94 °C for 30 s, annealing at 55 °C for 30 s, and extension at 72 °C for 30 s. This process concluded with a final extension at 72 °C for 2 min. The resulting PCR products were separated by electrophoresis on a 1% agarose gel [16]. The high-quality products were submitted to Sangon Biotech (Shanghai, China) Co., Ltd. for sequencing. The obtained sequences were subsequently compared against the NCBI database to identify the specific bacterial host species.

### 2.5. Characterization of Phages

#### 2.5.1. Transmission Electron Microscopy

The phage-concentrated solution was observed using transmission electron microscopy (TEM). The procedure began with the absorption of the phage particles onto a copper grid. This was followed by negative staining using 1% phosphotungstic acid at room temperature for 1–2 min. Finally, the phages on the copper grid were observed and imaged using TEM at an operating voltage of 80 kV [7].

#### 2.5.2. One-Step Growth Curve

The logarithmic-phase culture of the host bacteria and the phage-concentrated solution were inoculated into TSB, incubated at 37 °C for 10 min, and centrifuged at 7155× *g* at 4 °C for 5 min. The resulting precipitate was collected, washed thrice with TSB to remove free phages, and resuspended in 10 mL of TSB. The suspension was then cultured in a shaker at 37 °C. The phage titer was measured every 15 min using the double-layer plate method [17]. The supernatant was serially diluted in 10-fold increments to achieve an appropriate concentration. Aliquots of 100 μL of the diluted solution were mixed with 100 μL of logarithmic-phase host bacteria (10^8^ CFU/mL). Subsequently, 5 mL of semi-solid TSA (0.5% agar) was added to the mixture, gently mixed, and poured onto a pre-solidified TSA plate (1.5% agar). The plates were incubated at 37 °C for 24 h. The plaques were observed, and a one-step growth curve was established by correlating the logarithm of the phage titer (log PFU/mL) with the infection time. The latent period, burst period, and burst size of the phage were calculated based on the resulting data.

#### 2.5.3. Phage Tolerance Test

The isolated phages’ salt and acid tolerance were evaluated under specific conditions, a 4.5% salt concentration and pH levels of pH = 4.0, pH = 5.0, and pH = 6.0, respectively. For the salt tolerance evaluation, phage samples were incubated in TSB containing 4.5% salt for 4 h, followed by determining the phage titer using the double-layer agar plate method. Similarly, for acid tolerance testing, phage samples were incubated in TSB adjusted to pH = 4.0, pH = 5.0, and pH = 6.0 for 4 h each, with phage titers measured separately after each incubation. As a control, phage samples were incubated in untreated TSB for 4 h, and then their titers were determined.

### 2.6. The Impact of Phages ΦSx-2, ΦSs-1, and ΦSs-2 on the Fermentation of Sichuan Paocai

#### 2.6.1. Inoculating Phages into Sichuan *Paocai*

The red-skinned radishes purchased from a local market in Chengdu were thoroughly washed with tap water and air-dried at room temperature (20 ± 2 °C) under natural ventilation until no visible moisture remained on the surface. Subsequently, the radishes were cut into uniform pieces (approximately 4 cm in length). These pieces were submerged in glass jars containing brine with a final salt concentration of 4.5% in the fermentation system. Subsequently, phages ΦSx-2, ΦSs-1, and ΦSs-2 were inoculated into the jars at an inoculum size of approximately 10^8^ PFU. Finally, the jars were sealed by adding water to the rim and covered to allow natural fermentation at room temperature (20–25 °C). Simultaneously, a control group of Sichuan *paocai*, prepared without phage inoculation, was processed following the same procedures. Three jars of Sichuan *paocai* were ready for both the control group and the group inoculated with phages ΦSx-2, ΦSs-1, and ΦSs-2 (phages group), resulting in a total of six jars for parallel comparison. Sampling was conducted for physicochemical index measurements on the 0th, 7th, and 14th days of fermentation. Additionally, microbial diversity sequencing was conducted on the 0th, 7th, and 14th days of fermentation to analyze the bacterial community structure (sampling on the 0th day was conducted 4 h after phage inoculation).

#### 2.6.2. Determination of pH Value and Microbial Count of Sichuan *Paocai*

At each sampling time point, the pH value of brine samples was measured using a pH meter (PHS-3C, Fangzhou Technology, Fangzhou, China). The tryptic soy agar (TSA) and Man-Rogosa-Sharpe agar (MRS) supplemented with 1% (*w*/*v*) CaCO_3_ were used for total microbial count and lactic acid bacteria (LAB) count, respectively.

#### 2.6.3. Analysis of the Microbial Diversity of Sichuan *Paocai*

Microbial DNA was extracted from *paocai* brine samples on the 0th, 7th, and 14th days using the E.Z.N.A.^®^ Soil DNA Kit (Omega Bio-Tek, Norcross, GA, USA) following the manufacturer’s protocol. The V1-V9 region of the bacterial 16S rRNA gene was amplified by PCR using the primers 27F 5′-AGRGTTYGATYMTGGCTCAG-3′ and 1492R 5′-RGYTACCTTGTTACGACTT-3′. Amplicons were extracted from 2% agarose gels and purified using the AxyPrep DNA Gel Extraction Kit (Axygen Biosciences, Union City, CA, USA) according to the manufacturer’s instructions. SMRTbell libraries were prepared from the amplified DNA by blunt-end ligation. PacBio raw reads were processed using SMRT Link Analysis software (version 9.0) to obtain demultiplexed circular consensus sequence (CCS) reads. Subsequently, sequences were filtered to remove barcodes, primer sequences, chimeras, and any sequences containing 10 consecutive identical bases. OTUs were clustered with a 98.65% similarity cutoff using UPARSE (version 7.1, http://drive5.com/uparse/, accessed on 7 July 2023), and chimeric sequences were identified and removed using UCHIME.

The R stats package performed all statistical analyses. The relationships were visualized using correlation heatmaps from the R pheatmap v 1.0.12 package and network diagrams created with Cytoscape ver. 3.10.3 (http://www.cytoscape.org, accessed on 9 September 2023). One-way permutational analysis of variance (PERMANOVA) was performed using the R vegan package v 2.6-4 to evaluate the statistically significant effects of treatment processes on bacterial communities.

#### 2.6.4. Determination of Physicochemical Indexes of Sichuan *Paocai*

Hydrochloride naphthodiamide was used to measure nitrite concentrations [10]. The total acid was determined by titration with acid/base indicators [18]. The organic acid content in the *paocai* brine was analyzed using High-Performance Liquid Chromatography (HPLC). Lactic acid and acetic acid were separated using an Aminex HPX-87 H resin column (300 × 7.8 mm, Bio-Rad, Hercules, CA, USA), with 0.02 N sulfuric acid as the eluent and a flow rate of 0.6 mL/min [19].

#### 2.6.5. Flavor Determination and Color Observation of Sichuan *Paocai*

The Sichuan *paocai* brine was analyzed using an electronic tongue sensor for measurement. The measurement conditions were configured with a sensor amplification factor of 30×. The collected data were analyzed using linear discriminant analysis (LDA) software integrated with the electronic tongue system.

Color changes in the Sichuan *paocai* were recorded daily throughout the fermentation process.

### 2.7. Data Analysis

Three replicates of *paocai* fermentation were conducted for each container. All data presented are averages from at least three independent experiments. All statistical data were analyzed using one-way analysis of variance (ANOVA) with GraphPad Prism 9.0, where *p* < 0.05 was considered significant. Statistical significance levels were denoted as follows: **** *p* < 0.0001, *** *p* < 0.001, ** *p* < 0.01, and * *p*< 0.05, and “ns” for not significant. Graphical presentations were generated using Origin Software 8.0 and GraphPad Prism 7. All data analyses were performed using R v 4.3.1 [20] or Python ver. 3.13.2 (https://www.Python.org, accessed on 21 September 2023).

## 3. Results

### 3.1. Identification of Specific Hosts for Phages

In the experiment to isolate phages and their specific hosts, 94 bacterial strains were isolated as potential hosts for the phages. Using spot tests, 51 phage isolates were obtained, among which three were identified as phages with *Staphylococcus* as their host (Table 1).

### 3.2. Phage Characterization

#### 3.2.1. Phage Morphology

**Figure 1:**
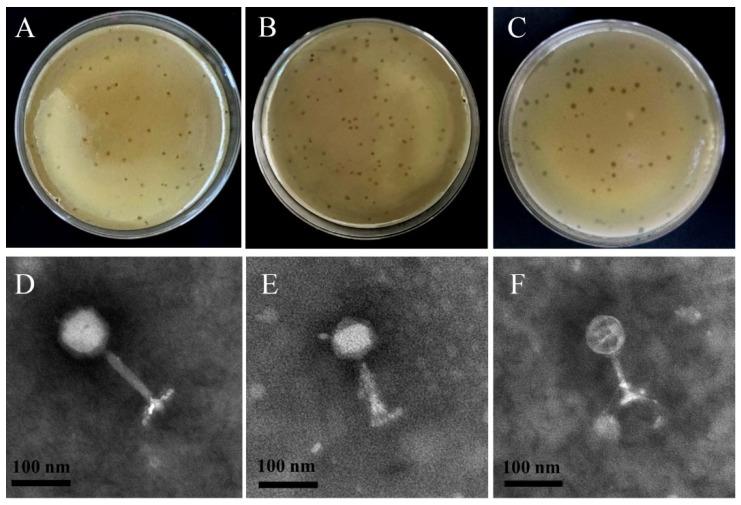
The plaque morphologies of phages ΦSx-2 (**A**), ΦSs-1 (**B**), and ΦSs-2 (**C**), as well as the transmission electron microscope images of phages ΦSx-2 (**D**), ΦSs-1 (**E**), and ΦSs-2 (**F**). Scale bar = 100 nm.

The phages ΦSx-2, ΦSs-1, and ΦSs-2 formed plaques with clear edges and uniform sizes on soft agar plates, demonstrating their ability to effectively lyse the host bacteria (Figure 1A–C). TEM images revealed that ΦSx-2, ΦSs-1, and ΦSs-2 possess icosahedral heads and contractile tails (Figure 1D–F). Based on the classification criteria of the International Committee on Taxonomy of Viruses (ICTV) [21], all three phages are classified in the Caudoviricetes class.

#### 3.2.2. One-Step Growth Curve of Phages

**Figure 2:**
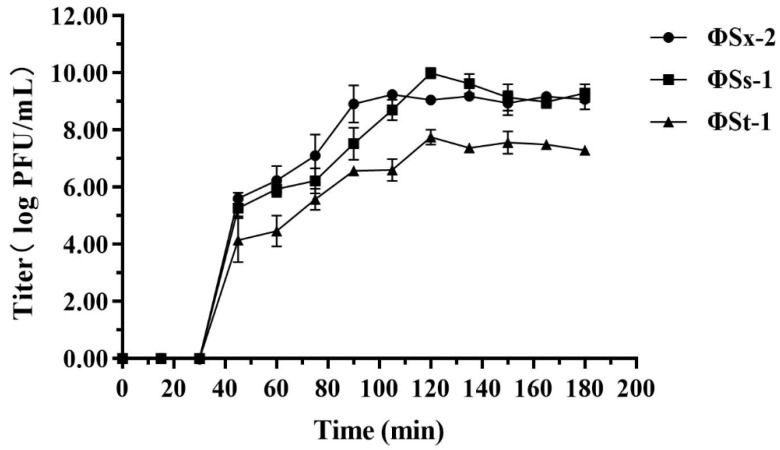
The one-step growth curves of phages ΦSx-2, ΦSs-1, and ΦSs-2 infecting their host bacteria in TSA medium (Circle: Growth curve of ΦSx-2; Square: Growth curve of ΦSs-1; Triangle: Growth curve of ΦSs-2). The values shown in the figures are the averages of three experiments.

The one-step growth curve results for phages ΦSx-2, ΦSs-1, and ΦSs-2 are shown in Figure 2. The latent periods for all three phages were approximately 30 min, followed by the lytic phase. For phage ΦSx-2, the lytic phase lasted approximately 60 min, with an average burst size of approximately 83 phage particles per infected cell. Phage ΦSs-1 had a lytic phase duration of approximately 90 min, with an average burst size of approximately 98 phage particles per infected cell. Similarly, the lytic phase for phage ΦSs-2 lasted around 90 min but with an average burst size of approximately six phage particles per infected cell.

#### 3.2.3. Analysis of Phage Tolerance

**Figure 3:**
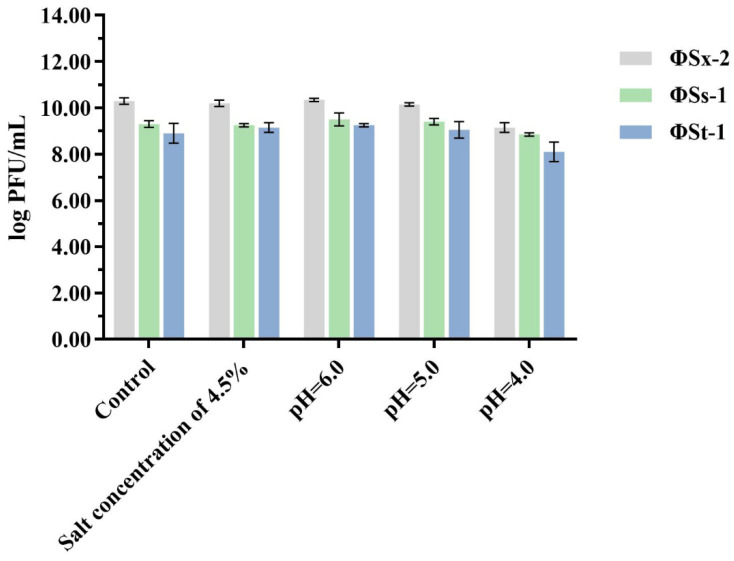
The survival of phages ΦSx-2, ΦSs-1, and ΦSs-2 under high salt concentration and low pH conditions. The values are the mean of three experiments, which showed no significant differences.

Dynamic monitoring of the Sichuan *paocai* fermentation process (Figure S1) revealed that the system’s pH value rapidly decreased from 6.0 to 4.5 during the first 3 days, dropped below the critical threshold of 4.0 by the 4th day, and stabilized around 3.5 by the 8th day, maintaining this level until the end of fermentation. Regarding salinity, although the initial brine was prepared at 6% (*w*/*v*), the actual salt concentration in the system was reduced to approximately 4.5% due to water released from vegetables through osmotic diffusion under the 1:3 vegetable-to-brine ratio. Building upon these measured parameters, we established a simulated fermentation system to investigate the effects of low pH (4.0–6.0) and moderately high salt (4.5%) environments on the activity of phages ΦSx-2, ΦSs-1, and ΦSs-2. Figure 3 shows the survival of phages ΦSx-2, ΦSs-1, and ΦSs-2 after exposure to environments with a salt concentration of 4.5% and pH levels of 6.0, 5.0, and 4.0 for 4 h, respectively. Compared to the control group, the activity of phages ΦSx-2, ΦSs-1, and ΦSs-2 remained essentially unchanged under these conditions, demonstrating their complete resistance. These experimental results indicated that phages ΦSx-2, ΦSs-1, and ΦSs-2 can effectively survive in the low-pH and high-salt fermentation environment of Sichuan *paocai*.

### 3.3. The Impact of Phages ΦSx-2, ΦSs-1, and ΦSs-2 on Sichuan Paocai Fermentation

#### 3.3.1. Changes in pH Value, Total Bacterial Count, and Lactic Acid Bacteria Count

**Figure 4:**
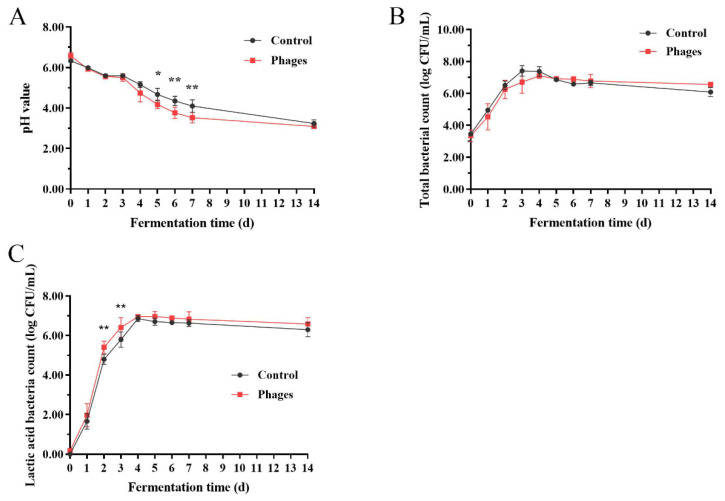
Changes in pH (**A**), total viable bacteria count (**B**), and lactic acid bacteria count (**C**) during the fermentation process of *paocai* (Control: *paocai* group without artificial inoculation of phages; Phages: *paocai* group with artificial inoculation of phages ΦSx-2, ΦSs-1, and ΦSs-2). Data are expressed as Mean ± SD (*n* = 3). (*) *p* < 0.05, (**) *p* < 0.01.

As shown in Figure 4A, during the fermentation process of Sichuan *paocai*, the pH value of both the control and phages groups decreased sharply from 6.5 to approximately 3.0. Notably, starting on the 4th day of fermentation, the pH value in the phages group declined more rapidly. The overall trends in total bacterial count and LAB count were similar for both groups: initially increased and then decreased. The total bacterial count peaked on the 3rd and 4th days of fermentation for the control and phages groups, respectively (Figure 4B). The LAB count for both groups peaked on the 4th day; however, throughout the entire fermentation process, the LAB count was consistently higher in the phages group than in the control group (Figure 4C).

#### 3.3.2. The Impact of Phages ΦSx-2, ΦSs-1, and ΦSs-2 on Microbial Community Structure

##### Alpha Diversity Indices of Different *Paocai* Samples

**Figure 5:**
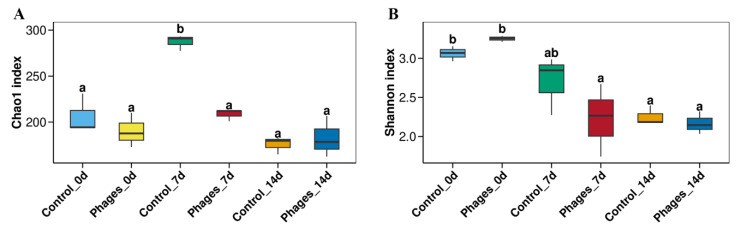
Temporal changes in alpha diversity indices during paocai fermentation. (**A**) Chao1 index showing community richness; (**B**) Shannon index indicating community diversity. Error bars represent standard deviations of triplicate biological replicates (*n* = 3). The letters a, b, etc., indicate the results of multiple comparison tests. Groups sharing the same letter are not statistically significant (*p* > 0.05), while groups with different letters show significant differences (*p* ≤ 0.05).

As shown in Figure S2, both the Shannon–Wiener index and the rarefaction curves tend to level off as sequencing depth increases, indicating that most bacterial taxa have been captured, meeting the requirements for subsequent bioinformatics analysis. The Alpha diversity indices of *paocai* fermentation are shown in Figure 5. The Chao1 index represents community richness, while the Shannon index reflects community diversity. The community diversity in both groups gradually decreased throughout the fermentation process. On the 7th day of fermentation, the community richness and diversity in the control group were significantly higher than in the phages group.

##### Analysis of Microbial Community Structure During *Paocai* Fermentation

**Figure 6:**
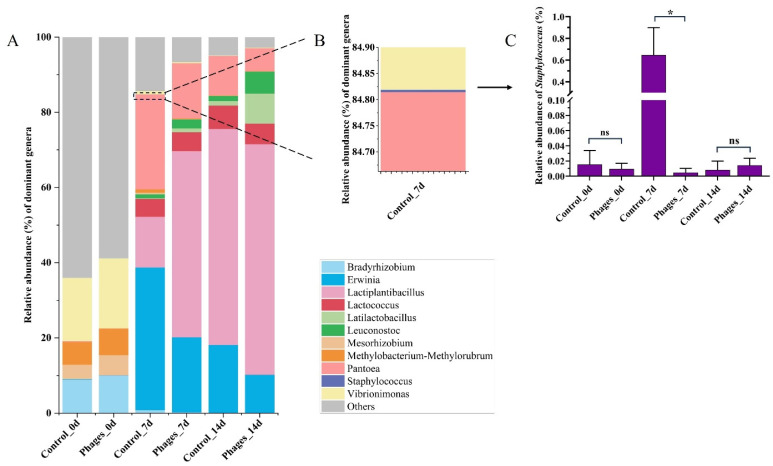
Microbial diversity composition on the 0th, 7th, and 14th days of fermentation (**A**) Stacked bar chart showing the bacterial community composition at the genus level; (**B**) Stacked bar chart illustrating the relative abundance of *Staphylococcus* in the control group on the 7th day of fermentation; (**C**) Relative abundance of *Staphylococcus* across different groups. Mean ± SD (*n* = 3). (ns) *p* > 0.05, (*) *p* < 0.05.

The sequencing results of bacteria collected on the 0th, 7th, and 14th days of *paocai* fermentation were annotated, with the characteristic microorganisms of each group shown in Figure S3. The top ten genera and *Staphylococcus* were selected to create stacked bar charts depicting the bacterial community composition (Figure 6A). On the 0th day of fermentation, the bacterial community composition was similar between the control and phages groups, with no significant difference in the proportion of *Staphylococcus* (Figure 6C). By the 7th day of fermentation, the abundance of *Staphylococcus* had increased significantly in the control group. The top three abundant genera in the control group at this stage were *Erwinia* (37.89%), *Pantoea* (25.27%), and *Lactiplantibacillus* (13.48%). In the phages group, *Lactiplantibacillus* was dominant, with a relative abundance of 49.54%, which was 36.08% higher than in the control group. By the 14th day, the relative abundance of *Staphylococcus* in the control group had decreased to levels comparable to the phages group. At this stage, *Lactiplantibacillus* (57.46%) became the most dominant genus in the control group, followed by *Erwinia* (18.05%) and *Pantoea* (10.49%). Meanwhile, in the phages group, *Lactiplantibacillus* (61.31%) remained the most dominant genus, with *Erwinia* (10.11%) and *Latilactobacillus* (7.98%) as the second and third most abundant genera, respectively.

##### Microecological Correlation Analysis

**Figure 7:**
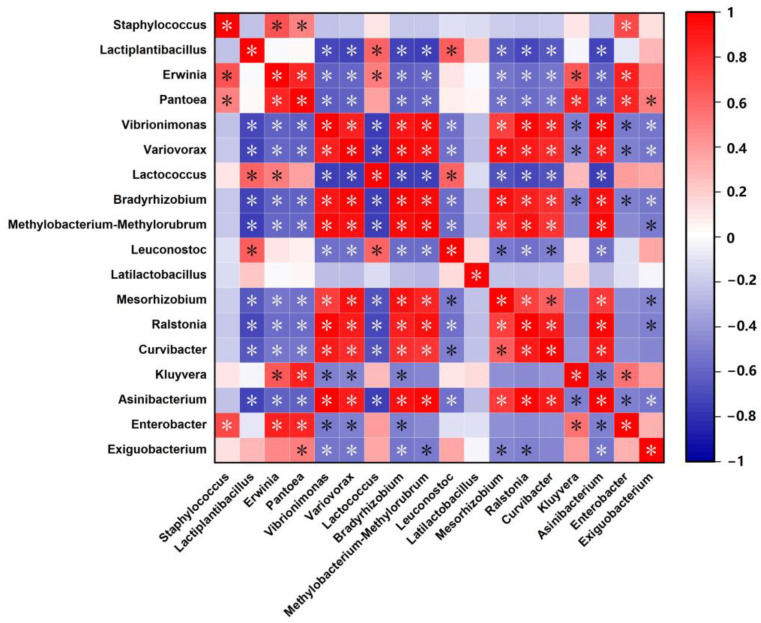
The correlation matrix mainly belongs to Spearman’s rank correlation between attributes. (*) *p* < 0.05.

The Spearman’s rank correlation method was applied to analyze the data between *Staphylococcus* and major bacterial genera, as shown in the correlation matrix in Figure 7. The results revealed that *Staphylococcus* exhibited a significant positive correlation with *Erwinia*, *Pantoea*, *Enterobacter*, and *Lactococcus*. In contrast, it showed a significant negative correlation with *Lactiplantibacillus* and *Leuconostoc*.

#### 3.3.3. Changes in Nitrite, Total Acid, and Organic Acid Contents

**Figure 8:**
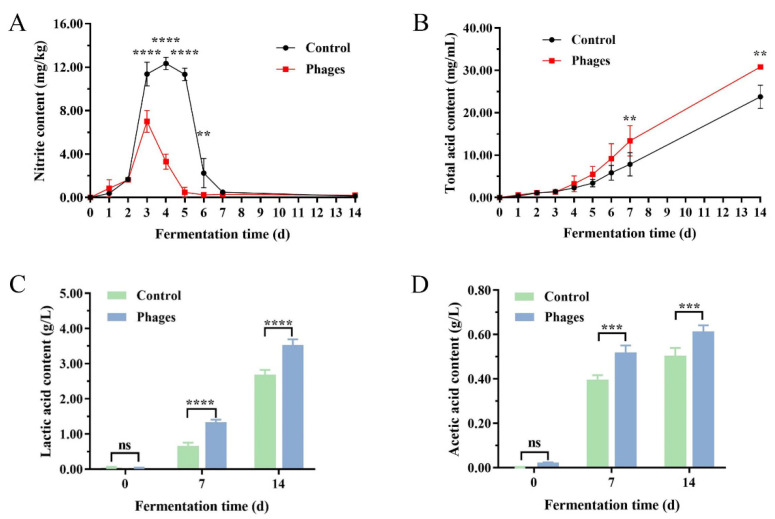
Changes in the contents of nitrite (**A**), total acid (**B**), lactic acid (**C**), and acetic acid (**D**) during the fermentation process of *paocai*. Data are expressed as Mean ± SD (*n* = 3), (ns) *p* > 0.05, (**) *p* < 0.01, (***) *p* < 0.001, (****) *p* < 0.0001.

As shown in Figure 8, the nitrite level in both groups initially increased and then decreased. In the control group, the nitrite level peaked at 12.50 mg/kg on the 3rd day of fermentation, while in the phages group, it peaked at 6.00 mg/kg on the 4th day. Both peak levels were below the national safety standard limit of 20 mg/kg [22]. By the 7th day of fermentation, the nitrite content in both groups had dropped to their lowest levels and remained stable thereafter (Figure 8A). The total acid content in both groups increased steadily throughout the fermentation process. From the 7th day onward, the total acid content in the phages group was significantly higher than in the control group (Figure 8B). Figure 8C,D shows that lactic acid and acetic acid levels increased significantly as fermentation progressed. On the 7th and 14th days of fermentation, the lactic acid content in the phages group was 0.681 g/L and 0.848 g/L higher, respectively, than in the control group. A similar trend was observed for the acetic acid content. Linear Discriminant Analysis (LDA) performed using an electronic tongue on the fermentation brine indicated that fermentation samples from both groups on the 0th, 7th, and 14th days exhibited unique aroma characteristics (Figure S4).

#### 3.3.4. Color Changes of Sichuan *Paocai* During Fermentation

Figure S5 illustrates the color changes of red-skinned radishes during the fermentation process. Starting from the 5th day of fermentation, both groups of pickled radishes began to develop a red hue, with the phages group displaying a noticeably deeper color. The changes in pH and LAB count indicate that the *paocai* in the phages group produced acid more rapidly, resulting in faster pigment extraction from the radish skin [23]. By the 7th day of fermentation, the radishes in the control group exhibited slight signs of browning, while those in the phages group displayed a more vibrant color. By the 14th day of fermentation, significant browning was evident in the control group, whereas the radishes in the phages group retained their bright and vivid color.

## 4. Discussion

Research on phages in various fermented foods has achieved significant advancements. However, in the context of traditional Chinese fermented vegetables, relevant research on phages remains relatively limited. This study addresses this gap by conducting a biological characterization analysis of phages isolated from Sichuan *paocai*. It explores their role in shaping the microbial community structure during the fermentation process and their impact on key physicochemical indicators. The findings contribute new insights to the knowledge system in this field and establish a foundation for further exploration and practical applications of phage potential in traditional Chinese fermented vegetable production.

This study classified three staphylococcal phages, ΦSx-2, ΦSs-1, and ΦSt-1, isolated from Sichuan *paocai* within the class Caudoviricetes. Consistent with currently available data, most phages infecting *Staphylococcus* are reported to belong to the Caudoviricetes order [24], which aligns with our results. The latent periods of phages ΦSx-2, ΦSs-1, and ΦSt-1 fell within the typical latent period for Caudoviricetes phages (21–120 min), and their lysis periods align with the lytic cycle dynamics previously reported for staphylococcal phages [25]. Additionally, the average burst sizes of ΦSx-2 and ΦSs-1 were within the typical lysis yield range (50–100 PFU/cell) observed for many Caudoviricetes phages [26]. Phages with large burst sizes (>50 PFU/cell) exhibit a selective advantage over competing phages, contributing to higher lytic activity [27]. Tolerance analysis revealed that low pH and high salt environments did not significantly affect the infectivity of phages ΦSx-2, ΦSs-1, and ΦSt-1. These findings suggest that these phages can survive and remain active in an environment with low pH and high salt concentrations of *paocai* fermentation.

Previous research has demonstrated that phages in fermented foods can significantly influence the product quality by lysis of hosts and regulating the metabolism of core microorganisms [28]. In this study, phages ΦSx-2, ΦSs-1, and ΦSt-1 were intentionally introduced as fermentation aids during the production of Sichuan *paocai*, ensuring direct interaction with the initial microbial community at the start of the fermentation. Key indicators for evaluating normal fermentation in Sichuan *paocai* include pH value, total bacterial count, and LAB count [29]. During fermentation, these parameters followed similar trends in both the control and phages groups. However, the pH dropped rapidly, and the LAB count was slightly higher in the phages group. A pH value below 4.0 indicates maturity, suggesting that the *paocai* in the phages group matured faster. Phages can accelerate the formation of an acidic environment, which is crucial for suppressing spoilage bacteria [15]. We speculate that these changes might be related to the phages’ ability to alter the microbial flora structure during fermentation.

The spontaneous fermentation system of vegetables exhibits a complex microbial structure with intricate interactions [30]. During the initial fermentation stage, the microbial community was primarily derived from the raw materials and containers [11]. Consequently, the microbial richness and diversity are initially similar between groups. By the 7th day, the *paocai* fermentation reached its midpoint, characterized by lactate accumulation, a decline in pH, and the dominance of LAB, resulting in increased richness but decreased diversity [18,31]. In the phages group, the number of *Staphylococcus* and spoilage bacteria (*Erwinia* and *Pantoea*) decreased, while the LAB population increased compared to the control group (Figure 6A). This shift explains the rapid pH drop and significant lactic acid accumulation observed in the phages group. In the later stages of fermentation, the control group was dominated by *Lactococcus* and *Weissella* species, whereas the phages group was dominated by *Lactiplantibacillus*, *Latilactobacillus*, and *Leuconostoc* species. Previous studies have demonstrated that *Lactobacillus* and *Leuconostoc* species emerge as the dominant core microbiota during the late-stage fermentation of radish pickles, playing pivotal roles in microbial community succession and flavor development [11]. Our findings fully align with this established conclusion. As fermentation progressed, the accumulation of lactic acid created an environment unsuitable for most microorganisms, further reducing microbial diversity. Meanwhile, the proliferation of LAB significantly influenced physicochemical indicators, particularly by markedly decreasing the peak nitrite level [32,33]. Previous studies have demonstrated that inoculating *Lactobacillus plantarum* ZJ316 effectively suppresses spoilage microorganisms such as *Pseudomonas* and *Enterobacteriaceae*, while promoting the development of probiotics such as *Lactobacillus* and *Bifidobacterium*, and also accelerates nitrite degradation in pickles [34]. Additionally, LAB continuously converted carbohydrates into organic acids, notably lactic and acetic acid, significantly increasing the total acid content [18,35]. These short-chain fatty acids contributed to nitrite degradation and played a key role in determining the flavor and quality of *paocai* [36,37,38]. Furthermore, lactic acid inhibited peroxidase activity, making the red-skinned radishes in the phages group less susceptible to oxidation after anthocyanin dissolution. This effect resulted in brighter colors and reduced browning [23,35].

Spearman correlation analysis revealed that the *Staphylococcus* genus was negatively correlated with various LAB genera and positively correlated with spoilage bacteria such as *Pantoea*, *Erwinia*, and *Enterobacter*. Based on these observations, the LAB content and the reduction in spoilage bacteria might be linked to the activity of phages targeting *Staphylococcus*. Research on intestinal microbiota has demonstrated that phages can indirectly shape intestinal microbial community structures by directly lysing bacterial hosts, thereby significantly affecting human health [39]. The dynamic equilibrium within microbial communities’ microecological network suggests that even minor perturbations can lead to intricate and unpredictable outcomes [40]. Consequently, we inferred that phages might indirectly modulate microbial community composition by lysing *Staphylococcus* hosts, leading to observable variations among these communities [39]. Additionally, phages not only directly interact with their targeted host bacteria but also indirectly influence non-targeted microbial populations through ecological regulation [41]. This cascade effect demonstrates that phage application can significantly alter the community structure and relative abundance of both low- and high-abundance microorganisms in kimchi and other fermentation systems, including those bacterial taxa that are intrinsically insensitive (non-host) to the phages themselves [41]. Furthermore, phages play a role in modifying competitive dynamics among bacterial strains or species, thereby contributing to the maintenance of bacterial diversity to some extent [42].

Although the introduction of phages did not significantly disrupt the microbial community structure, it resulted in minor, generally beneficial changes. The nitrite level decline and the LAB increase suggest a shift toward a more probiotic-friendly profile in Sichuan *paocai*. In recent years, extensive research has focused on controlling nitrite content and preventing spoilage in vegetable fermentation, with phages emerging as a novel regulatory strategy to address these challenges. This study represents an initial exploration of this potential, demonstrating that phages can influence the microbiome of the Sichuan *paocai* fermentation system. It addresses a critical knowledge gap and provides a foundation for further investigation and practical application of phage technology in fermented foods. However, due to the limited duration of this experiment, we could not observe the long-term effects of microbiome alterations on the subsequent fermentation process of Sichuan *paocai*. Future studies will investigate the role of phage-induced microbiome structural changes in improving the quality and stability of fermented vegetables over extended fermentation periods.

## 5. Conclusions

This study isolated three staphylococcal phages (ΦSx-2, ΦSs-1, and ΦSs-2) from Sichuan *paocai* and systematically investigated their effects on microbial communities and quality during fermentation. The results demonstrated that phage introduction significantly increased the relative abundance of LAB while effectively inhibiting the growth of spoilage bacteria (e.g., *Pantoea*, *Erwinia*, and *Enterobacter*). This optimized microbial community structure accelerated the acidification process, reducing nitrite content and increasing lactic acid and acetic acid production, while significantly improving the color and flavor characteristics of *paocai*. Although these findings demonstrate the potential of phages in optimizing fermentation processes, further exploration is still required. Future research should focus on elucidating the interaction mechanisms between phages and microbial communities and their effects on metabolic pathways. Additionally, optimizing phage cocktails (e.g., combining multiple phages or adjusting their ratios) may enhance specificity and efficacy against target pathogens while preserving beneficial microbiota. By addressing these key issues, phage technology could become a green control strategy for traditional fermented food production.

## Figures and Tables

**Table 1:** Phages that form turbid pinpoint plaques and their corresponding hosts.

Phage	Host		
ID	ID	ID by 16S rRNA Sequencing	% Identity/Coverage
ΦSx-2	Sx-2	*Staphylococcus xylosus*	99.36/99
ΦSs-1	Ss-1	*Staphylococcus sciuri*	99.23/99
ΦSs-2	Ss-2	*Staphylococcus sciuri*	98.25/99

The host identification shown in this table is based on 16S rRNA gene sequencing.

## Data Availability

The authors declare that the data supporting the findings of this study are available within the paper and its Supplementary Information Files. Should any raw data files be needed in another format, they are available from the corresponding author upon reasonable request.

## Supplementary Materials

The following supporting information can be downloaded at: https://www.mdpi.com/article/10.3390/microorganisms13061273/s1.
