# Supplementary material for: Isolation, Characterization, and Preliminary Application of Staphylococcal Bacteriophages in Sichuan Paocai Fermentation
Source: Microorganisms. 2025 May 30;13(6):1273. doi: 10.3390/microorganisms13061273 (PMC12195502; doi:10.3390/microorganisms13061273)
Supplement: Supplementary file 1 [file microorganisms-13-01273-s001.zip › microorganisms-3640038-supplementary.pdf]

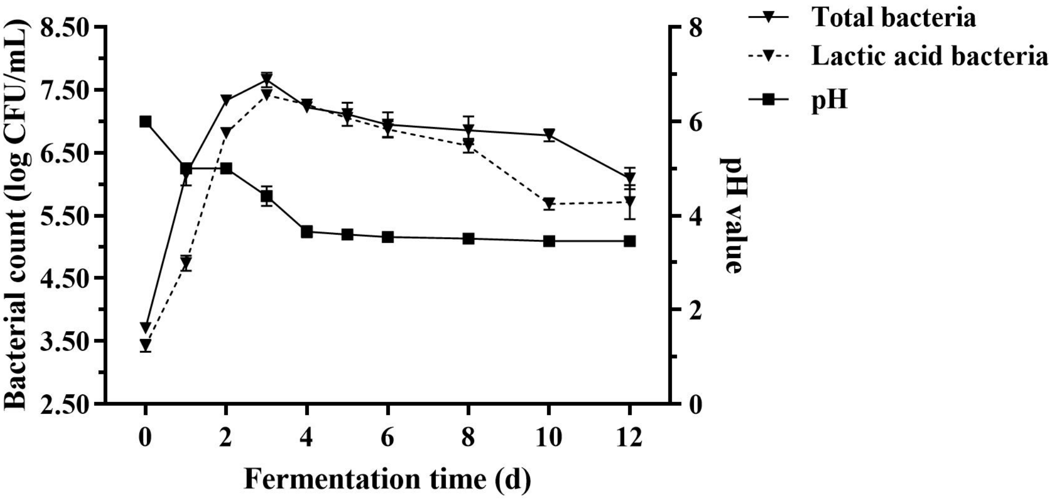

Figure S1. Changes in pH, total bacterial count, and lactic acid bacteria population during the fermentation of Sichuan *paocai*.

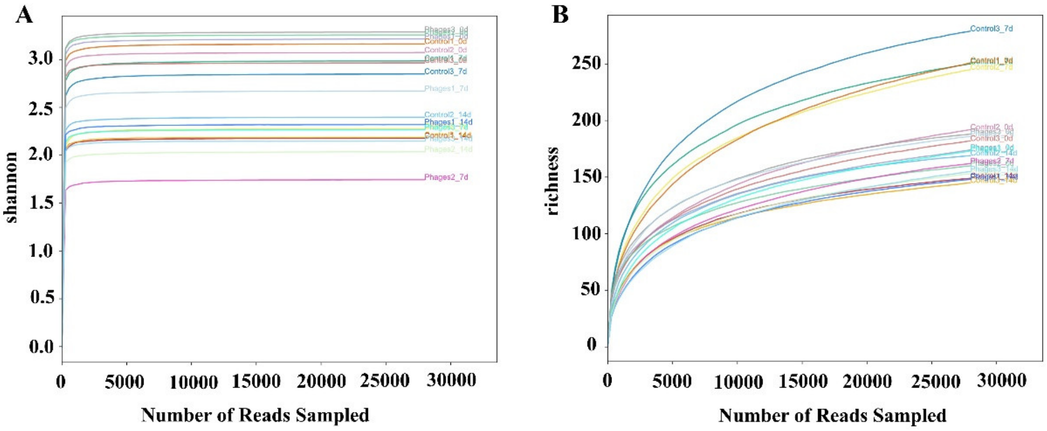

Figure S2. Evaluation of sequencing depth for microbial community analysis. (A) Saturation curves of Shannon-Wiener index; (B) Rarefaction curves of observed OTUs at different sequencing depths.

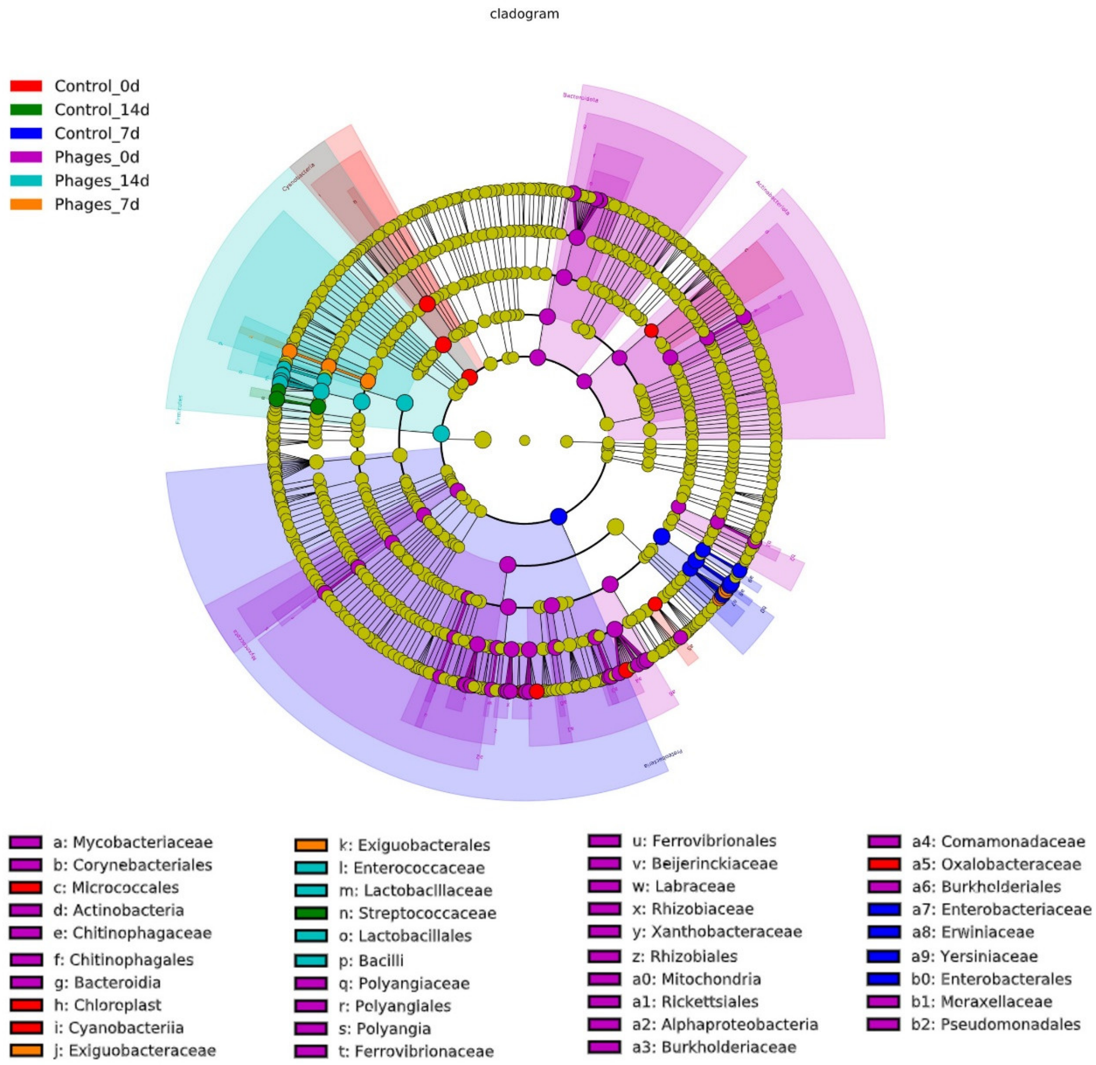

Figure S3. The LEfSe analysis of microbial communities on the 0th, 7th, and 14th days of fermentation.

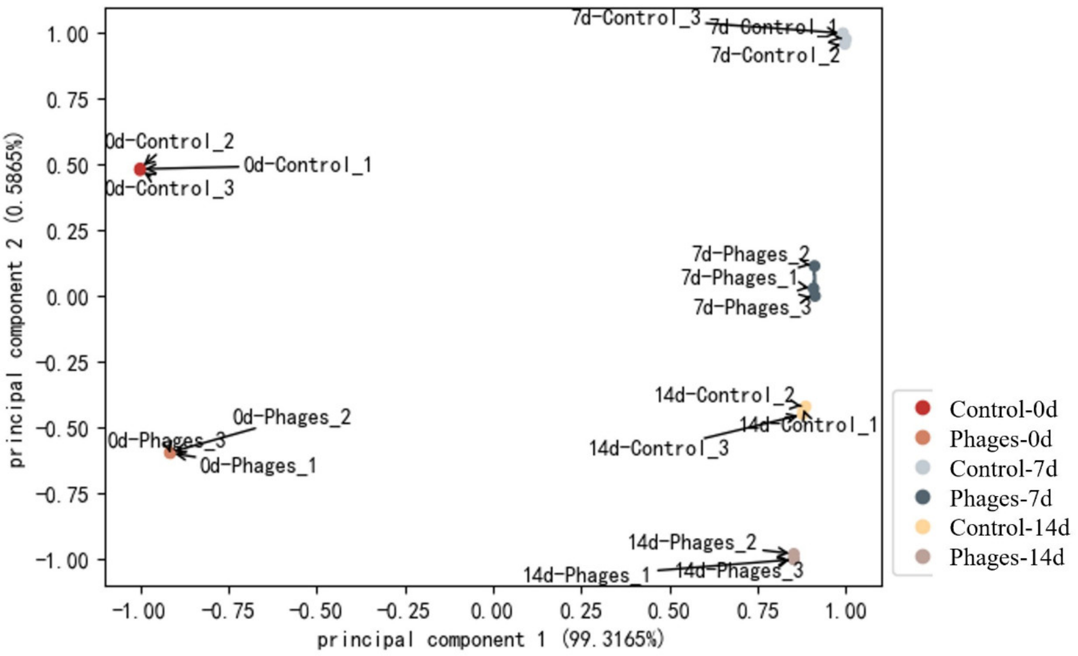

Figure S4. Linear Discriminant Analysis (LDA) results of the electronic tongue for Sichuan *paocai* on the 0th, 7th, and 14th day of fermentation.

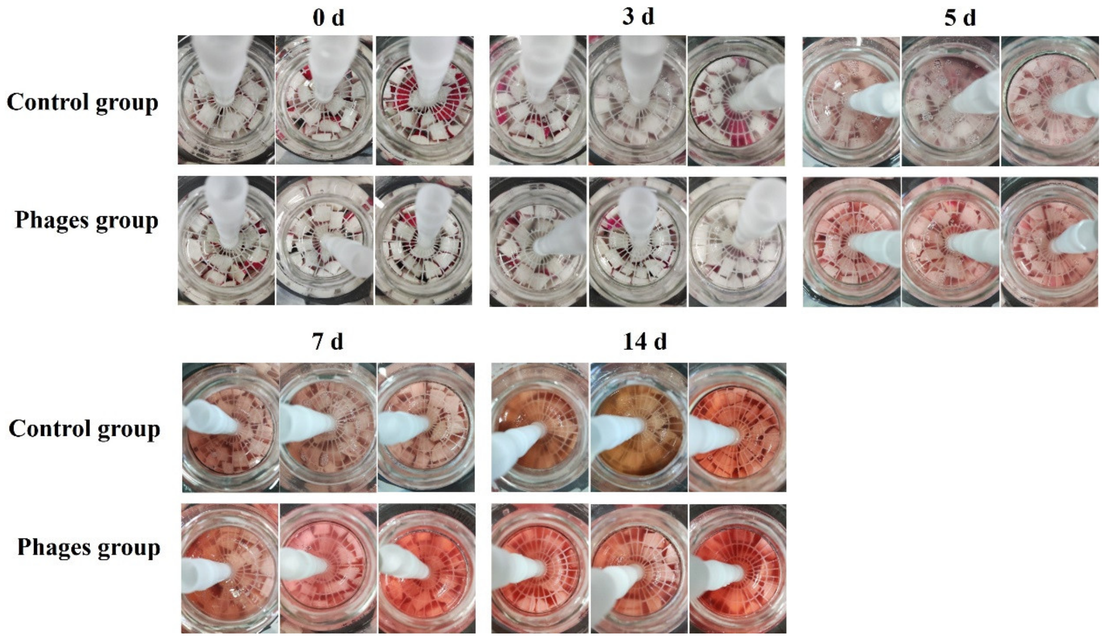

Figure S5. The color changes during the fermentation process of Sichuan *paocai* (using red-skinned radishes as the raw material).
